# Towards Effective and Socio-Culturally Appropriate Sanitation and Hygiene Interventions in the Philippines: A Mixed Method Approach

**DOI:** 10.3390/ijerph120201902

**Published:** 2015-02-05

**Authors:** Lisa Maria Pfadenhauer, Eva Rehfuess

**Affiliations:** Institute for Medical Informatics, Biometry and Epidemiology, University of Munich, Marchioninistrasse 15, 81377 Munich, Germany; E-Mail: rehfuess@ibe.med.uni-muenchen.de

**Keywords:** water, sanitation and hygiene (WASH), anal cleansing behaviour, demographic and health survey (DHS), mixed methods, focus group discussion, thematic analysis, Philippines, developing countries, complex interventions

## Abstract

Inadequate water, sanitation and hygiene (WASH) represent an important health burden in the Philippines. The non-governmental organisation Fit for School intends to complement its handwashing programme in schools with sanitation interventions. The objectives of this mixed-method study therefore were to describe WASH practices and their impact on childhood diarrhoea in the Philippines, and to examine socio-cultural and environmental factors underlying defecation and anal cleansing practices in Northern Mindanao. We quantified the effect of WASH practices on diarrhoea through logistic regression models, using the Philippine Demographic and Health Survey 2008. When adjusting for non-modifiable factors, susceptibility and socioeconomic factors, WASH factors failed to show a statistically significant effect. Focus group discussions were held with women in urban and rural Northern Mindanao, and findings analysed using thematic analysis. Defecation and anal cleansing behaviours were constrained by the physical environment, particularly the lack of clean, safe, comfortable and private facilities. Individual determinants of behaviour were influenced by habit and motivations such as disgust, with some evidence of planned behaviour. Where available, water was the preferred material for anal cleansing. This study combines nationally-representative quantitative data with local in-depth qualitative insights, constituting critical formative research in the development of effective and appropriate interventions.

## 1. Introduction

Globally, diarrhoea is the fourth leading cause of death, and claimed 2.5 and 1.4 million lives in the years 1990 and 2010 respectively [1]. A major share of these deaths is attributable to water, sanitation and hygiene (WASH) [2]. Indeed, together, unimproved water and sanitation are estimated to be responsible for 2.1% of disability-adjusted life years (DALYs) in the year 1990, and for 0.9% of DALYs in 2010 [3]. Systematic reviews have shown that water supply interventions can reduce the burden of disease by 25% to 27% [4,5], water quality interventions by 17% to 42% [4,5,6,7], while sanitation can achieve a risk reduction from 22 to 37% [4,5,6,7]. Estimates for the effectiveness of hand washing range from 31% to 48% [6,7,8,9].

In the Philippines, the burden imposed by WASH accounts for 15,000 annual deaths [10]. In 2010, 26% of the population had no access to improved sanitation facilities (urban: 21%, rural 31%), while 8% had no access to improved water sources (urban: 7%, rural: 8%) [11]. According to recent estimates, in three out of the five regions of Mindanao, the second largest island of the Philippines, more than 20% of children rely on unimproved sanitation [12].

Providing appropriate water and sanitation technology is not sufficient for effective diarrhoea prevention: accompanying behavioural changes are crucial, if long-term acceptability and sustained use are to be achieved. Diverse theories exist about how behaviour is determined and influenced, including the Health Belief Model [13], the Theory of Reasoned Action [14] and the Theory of Planned Behaviour [15]. Drawing on recent findings from psychology and neuroscience [16,17,18,19], Curtis and colleagues developed a conceptual model which suggests that individual (e.g., habitual, planning and motivational) causes, combined with environmental (e.g., biological, physical and social environment) causes, shape handwashing behaviour [20]. Interventions must be designed to affect both sets of causes. Children suffer from the highest WASH-attributable health burden [3]: while children under five years of age are most vulnerable to poor WASH facilities and practices at home, older children who spend more time in schools, are affected by a lack of appropriate facilities in this setting. Therefore, both homes and schools are critical in creating conducive conditions for healthy behaviours as well as promoting appropriate sanitation and hygiene behaviours through knowledge, attitudes and skills [21]. Women are usually responsible for maintaining sanitation facilities, educating children about sanitation and hygiene and looking after children with diarrhoea. Understanding their perceptions, constraints and motivations for change is therefore critical in the development of socio-culturally appropriate interventions.

The Philippino non-governmental organisation Fit for School and its partner, the German Development Cooperation (GIZ), have been promoting simple daily hygiene practices—handwashing and brushing teeth—as well as bi-annual deworming in more than 7000 elementary schools across the country for several years. Fit for School is currently considering the addition of further WASH elements to its school programme to improve child health and to encourage school attendance. In this general context, this mixed-method study was conducted as formative research to support the development of appropriate sanitation and anal cleansing interventions in schools and in the community. The interaction between technical and behavioural components of WASH as well as between family and school practices lie at the heart of our interest: our objectives were to understand national WASH practices in the Philippines and their impact on childhood diarrhoea, and to examine local socio-cultural and environmental factors underlying sanitation and anal cleansing practices in the region of Northern Mindanao.

## 2. Methods

### 2.1. Quantitative Methods

**Figure 1:**
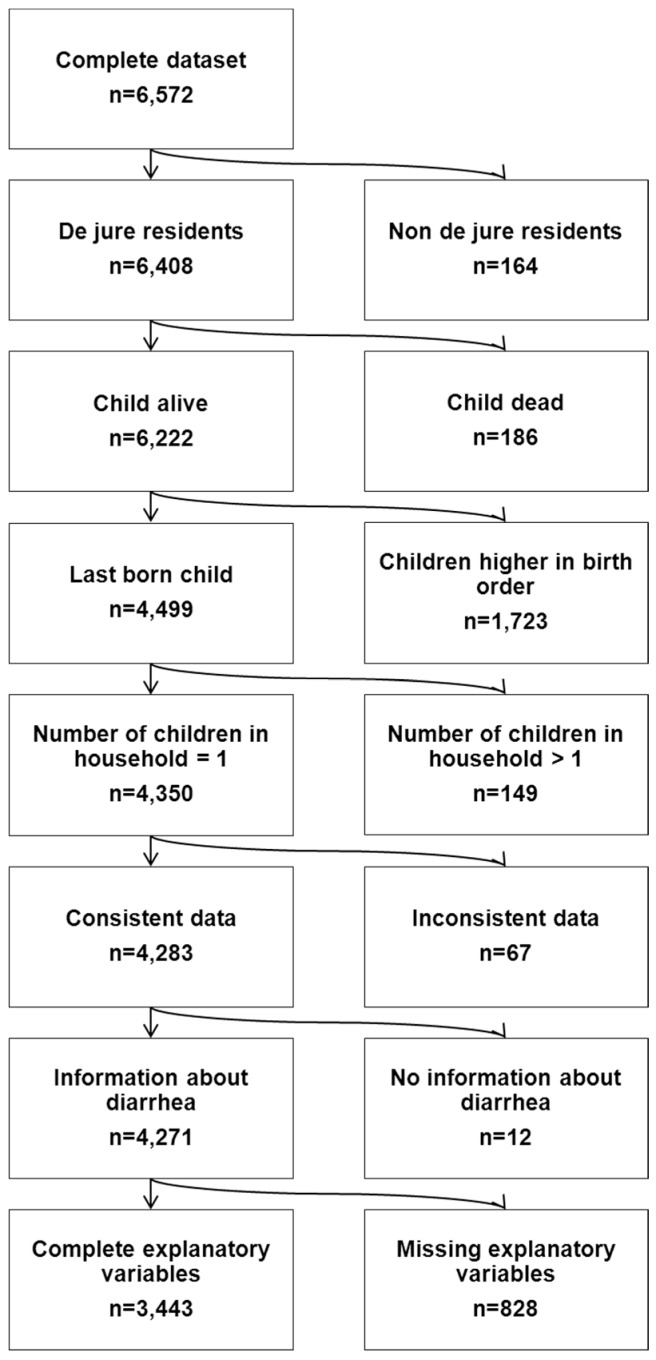
Flow chart of study population selection process.

The quantitative component of this study aimed to assess the influence of WASH practices and other risk factors on childhood diarrhoea in the Philippines. To do so, we used data from the Philippine Demographic and Health Survey (DHS) conducted in 2008, in which a nationally representative sample of 13,594 women in 12,469 households were interviewed [22]. Using a standardized three-staged probability sampling scheme this survey provides information on population, family planning and health; details of the sampling procedures and characteristics of the survey are published elsewhere [22]. Our study population was defined as the youngest child (as multiple children per household would result in non-independent observations) living as a de jure resident in a given household (as several of the factors influencing diarrhoea risk are household characteristics), and comprised 4350 children under five years of age. 67 observations were removed due to inconsistent data on number of children living in the household, a further 12 due to missing information on the outcome of interest and 828 observations due to missing information in explanatory variables, resulting in a final sample of 3443 children (Figure 1).

Our outcome of interest was the occurrence of watery stool among children younger than 60 months during the two weeks preceding the survey, as reported by the mother. We developed a list of all relevant diarrhoea risk factors as described by the World Health Organization (WHO) [23], complemented with literature searches in PubMed. The DHS dataset was screened for these factors to identify corresponding variables (e.g., maternal education) or proxies (e.g., distance to water source as a surrogate for water quantity). For some risk factors (e.g., handwashing) no corresponding variables were identified. Coding categories are either based on answer categories as defined by the DHS or on standard classifications (e.g., WHO/UNICEF Joint Monitoring Programme (JMP)) and provided in Table 1. The WASH complex comprised four variables: water source (defined as piped, improved, unimproved or surface water according to the WHO/UNICEF JMP [24]), distance to water source (on premises or delivered, less than five minutes, five minutes or more according to WHO [25] with five minutes also representing the median), sanitation facilities (improved, shared improved, unimproved, open defecation according to the WHO/UNICEF JMP [24]) and stool disposal (sanitary such as rinsing children’s faeces in toilets *versus* unsanitary such as disposing faeces in rivers according to WHO/UNICEF [26]). All variables shown in Table 1 were examined for collinearity; for any two showing a Spearman’s correlation coefficient of >0.6 one variable was excluded.

The impact of all explanatory variables was assessed using univariable logistic regression; interactions between selected variables were also explored. Subsequently, a multivariable logistic regression model was applied to examine how the WASH complex impacted diarrhoea changes, as one increasingly adjusts for confounders and competing risk factors. We entered each pre-defined set of covariates stepwise, starting with a simple model containing the WASH complex (model 1: water source, distance to water source, sanitation, stool disposal), then adjusting for non-modifiable characteristics (model 2: child age, sex, twin, region), susceptibility to diarrheal diseases based on a child’s nutritional and immune status (model 3: iron and vitamin A supplementation, intestinal parasite medication, breastfeeding, vaccination, including an interaction term for vaccination and child age) and socio-economic characteristics (model 4: household wealth, maternal characteristics, religion). Following O’Donnell *et al.*’s recommendation not to adjust for survey design effects as a conservative strategy for multivariable analysis of household survey data, we did not adjust for stratification and cluster samples and omitted sample weights [27].

Given the large number of missing values for vitamin A supplementation, we conducted a sensitivity analysis (SA) without this variable, yielding a sample of 4112 children.

While not used in model selection, we assessed the goodness of fit of each model with Nagelkerke’s *R*^2^, a pseudo *R*^2^ measure. All analyses were undertaken in SPSS v20.0 [28].

**Table 1:** Risk Factors according to diarrhoea occurrence during two weeks preceding the survey (*N* = 3443 children).

Risk Factor		No Diarrhea (*N* = 3091, 89.78)		Diarrhea (*N* = 352, 10.22)	
		*N*	%	*N*	%
**Water, Sanitation and Hygiene (WASH)**					
Sanitation	Improved	1888	61.08	193	54.83
	Improved, shared	732	23.68	76	21.59
	Unimproved	137	4.43	29	8.24
	Open defecation	334	10.81	54	15.34
	Total	3091	100.00	352	100.00
Disposal of Children’s Stool	Sanitary	1758	56.87	159	45.17
	Unsanitary	1333	43.13	193	54.83
	Total	3091	100.00	352	100.00
Water Source	Piped	842	27.24	80	22.73
	Improved	1371	44.35	183	51.99
	Unimproved	863	27.92	87	24.72
	Surface	15	0.49	2	0.57
	Total	3091	100.00	352	100.00
Distance to Water Source	On premises or delivered	2096	67.81	212	60.23
	<5 Min	521	16.86	65	18.47
	≥5 Min	474	15.33	75	21.31
	Total	3091	100.00	352	100.00
**Non-Modifiable Characteristics**					
Child Age (months)		Mean: 27.43; Median: 25.00, SD: 16.02; Range: 1.00–59.00		Mean: 20.91; Median: 18.00; SD: 11.87; Range: 2.00–58.00	
Child Sex	Female	1460	47.23	159	45.17
	Male	1631	52.77	193	54.83
	Total	3091	100.00	352	100.00
Twin	Single birth	3062	99.06	351	99.72
	Multiple birth	29	0.94	1	0.28
	Total	3091	100.00	352	100.00
Region	Ilocos Region	157	5.08	17	4.83
	Central Luzon	136	4.40	12	3.41
	Bicol Region	243	7.86	30	8.52
	Western Visayas	199	6.44	13	3.69
	Central Visayas	199	6.44	35	9.94
	Eastern Visayas	203	6.57	22	6.25
	Zamboanga Peninsula	156	5.05	20	5.68
	Northern Mindanao	151	4.89	20	5.68
	Davao Peninsula	154	4.98	9	2.56
	SOCCSKSARGEN	174	5.63	10	2.84
	Caraga	139	4.50	33	9.38
	National Capital Region	171	5.53	20	5.68
	Cordillera Admin Region	328	10.61	34	9.66
	ARMM	134	4.34	10	2.84
	CALABARZON	105	3.40	21	5.97
	MIMAROPA	299	9.67	27	7.67
	Total	143	4.63	19	5.40
Residence	Urban	1365	44.16	147	41.76
	Rural	1726	55.84	205	58.24
	Total	3091	100.00	352	100.00
**Susceptibility**					
Breastfeeding Status	Never breastfed	290	9.38	28	7.95
	Ever breastfed	2801	90.62	324	92.05
	Total	3091	100.00	352	100.00
Vitamin A Supplementation (past 6 months)	No	520	16.82	54	15.34
	Yes	2571	83.18	298	84.66
	Total	3091	100.00	352	100.00
Iron Supplementation	No	1869	60.47	223	63.35
	Yes	1222	39.53	129	36.65
	Total	3091	100.00	352	100.00
Intestinal Parasite Medication (past 6 months)	No	1975	63.90	247	70.17
	Yes	1116	36.10	105	29.83
	Total	3091	100.00	352	100.00
Vaccination Index (cumulative recommended vaccine shots against BCG (1), DPT(3), polio(3) and measles (1))	Low (0–2)	148	4.79	18	5.11
	Intermediate (3–5)	256	8.28	37	10.51
	High (6–8)	2687	86.93	297	84.38
	Total	3091	100.00	352	100.00
**Socioeconomic Characteristics**					
Maternal Age (years)		Mean: 30.53; Median: 30.00; SD: 6.84; Range: 15.00–49.00		Mean: 29.41; Median: 29.00; SD: 6.62; Range: 16.00–49.00	
Maternal Education	None or primary	724	23.42	109	30.97
	Secondary	1463	47.33	165	46.88
	Higher	904	29.25	78	22.16
	Total	3091	100.00	352	100.00
Maternal Working Status	Not working	1711	55.35	204	57.95
	Working	1380	44.65	148	42.05
	Total	3091	100.00	352	100.00
Household Wealth Index (composite measure based on asset ownership; households are grouped in quintiles)	Poorest	788	25.49	118	33.52
	Poorer	707	22.87	96	27.27
	Middle	616	19.93	62	17.61
	Richer	569	18.41	43	12.22
	Richest	411	13.30	33	9.38
	Total	3091	100.00	352	100.00
Religion	Roman Catholic	2407	77.87	265	75.28
	Protestant	154	4.98	25	7.10
	Islam	159	5.14	16	4.55
	Other	371	12.00	46	13.07
	Total	3091	100.00	352	100.00
Number of household members		Mean: 5.96; Median: 6.00; SD: 2.20; Range: 2.00–19.00		Mean: 6.02; Median: 6.00; SD: 2.43; Range: 2.00–18.00	
Number of under five children in household		Mean: 1.55; Median: 1.00; SD: 0.71; Range: 1.00–7.00		Mean: 1.65; Median: 2.00; SD: 0.76; Range: 1.00–6.00	

### 2.2. Qualitative Methods

The qualitative component of this study used six focus group discussions (FGD) with adult women to examine socio-cultural and environmental factors underlying sanitation and anal cleansing practices in urban and rural areas of Northern Mindanao. This region was chosen due to the long-standing activities of fit for school and plans to expand the ongoing school programme on handwashing and brushing teeth with a sanitation component. All FGDs were undertaken in November 2011.

Adopting a convenience sampling approach, we took advantage of weekly mother classes held in the city of Cagayan de Oro (4 FGDs) and in the villages Initao and Salay located close to Cagayan (1 FGD each), where women learn about gardening, crafting and specific health issues such as family planning. We initially approached the health workers conducting these classes, who all agreed to participate in the study. These mother classes are characterised by trust, an important precondition for frank but confidential discussions. Participation in mother classes is open to all community members and, to avoid any disruption of these regular processes, we imposed no restrictions on participation in the FGDs. Sites for the FGDs were the mothers' houses in Cagayan and open air assemblies in the two villages.

We developed an FGD guide comprising open-ended questions and probing questions (only applied when needed), with sections addressing current sanitation, personal hygiene and handwashing facilities and behaviours, current hygiene practices and education of babies and young children and aspirations for improved sanitation. The FGD guide was piloted in the first group, where it emerged that there was no need for changes. Verbal informed consent was obtained from all participants and recorded. The FGDs were conducted in Visaya, the local dialect, by a female local health worker, well-known among the groups. Data were audio-recorded by LMP (4 FGDs) and Fit for School staff (2 FGDs); notes from the FGDs were taken by female Fit for School (3 FGDs) and German Doctors staff (3 FGDs). The duration of the FGDs ranged from 45 to 90 min. LMP was at that time a Master student (Public Health), doing a 6 month research internship with Fit for School. In those FGDs, where LMP was present, she was introduced to participants by the interview moderator. Data saturation, which was discussed with the health workers involved in the interviews, was achieved after five FGDs, thus rendering the sixth FGD a mainly confirmative discussion. Data were transcribed orthographically in Visaya and translated into English by a researcher from Xavier University in Cagayan de Oro. These transcripts were checked by the health worker who conducted the interviews. They represent a verbatim record of what participants said, including non-verbal utterances.

**Figure 2:**
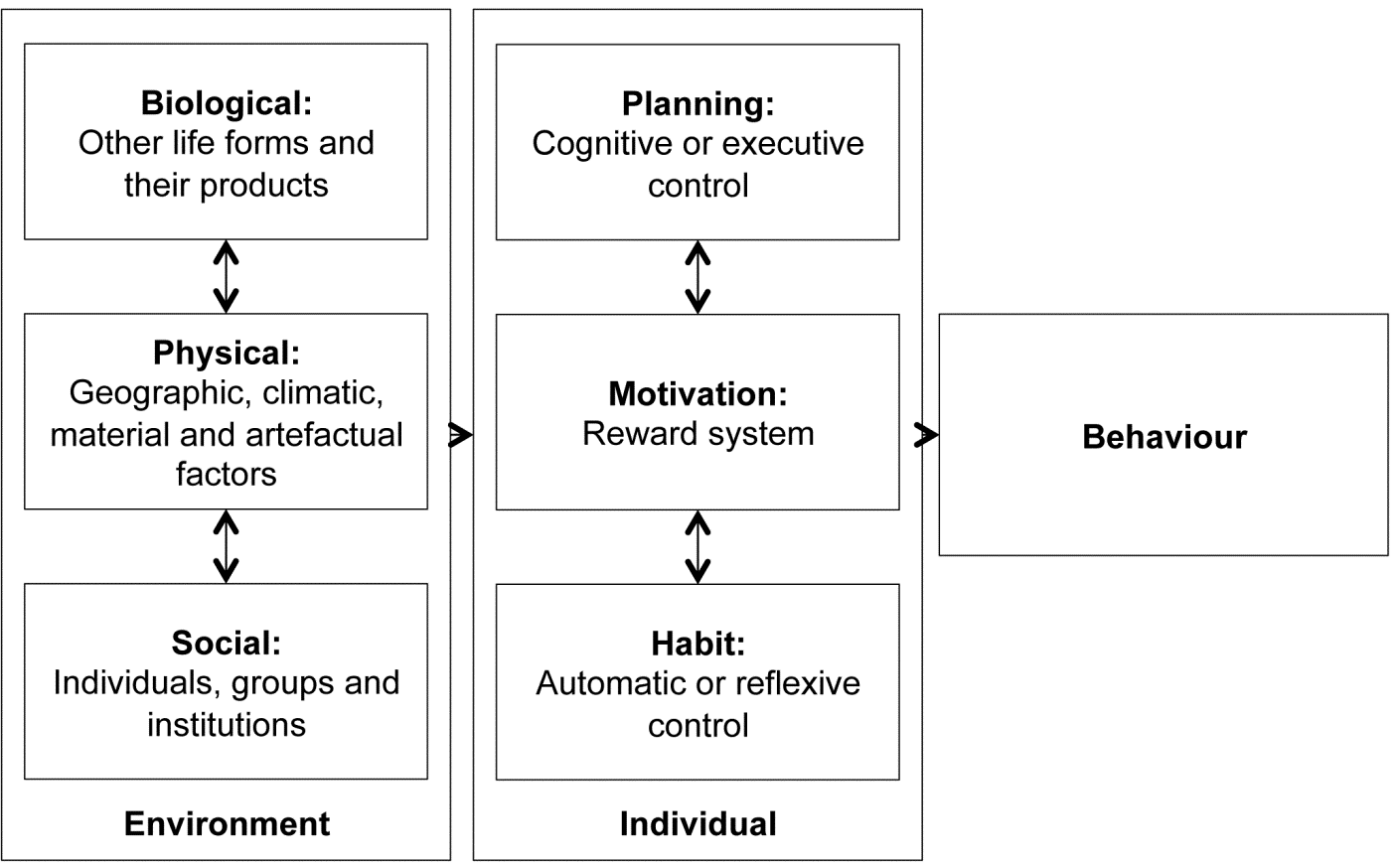
Conceptual framework of the determinants of defecation and anal cleansing behaviours.

We used thematic analysis in an inductive, data-driven way [29]. The analysis was performed manually by LMP, with EAR being consulted on a regular basis. After applying line-by-line coding to all six transcripts, initial descriptive themes were generated. Consecutively, these themes were refined into analytical themes in an iterative process involving interpretation. We paid attention to differential findings for rural and urban groups, as well as statements made in relation to children. To facilitate comparability, we adapted and extended Curtis’ conceptual model of the causes of handwashing behaviour to address the determinants of defecation and anal cleansing behaviour (Figure 2, adapted from [20]). This was used to develop higher-order categories and to embed our findings in a recognised framework.

The inter-subjectivity of the research process was reflected during data collection and analysis. As cultural outsiders, both researchers are naturally challenged with assumptions, etic perceptions and stereotypes.

### 2.3. Integration of Findings

To integrate findings and to achieve a more in-depth understanding of the issues around sanitation and anal cleansing practices, we followed the triangulation protocol as described by Farmer *et al.* [30]. The approach sets out to identify meta-themes across the findings from different methods, while specifically looking at agreement, partial agreement, silence, or dissonance between findings from different components [31]. To do so, a convergence coding matrix was created, contrasting findings from the qualitative and quantitative components while specifically focusing on inter-method discrepancies. Integrated findings were presented following the structure of the quantitative analysis.

### 2.4. Ethic Statement

For this study, ethical approval was not required since—except for age—no personal data were gathered.

## 3. Results

### 3.1. Quantitative Component

This section is concerned with the quantitative impact of multiple risk factors of childhood diarrhoea in the Philippines. While most variables showed a low level of collinearity; maternal and paternal education and age were highly correlated with Spearman’s correlation coefficients of 0.61 and 0.76 respectively. Consequently; paternal education was not considered in multivariable analyses. Table 1 depicts the distribution of all risk factors with respect to diarrhoea status.

While type of water source and distance to water source do not show statistical significance in any of the models, overall, the multivariable logistic regression analyses suggest that unimproved sanitation facilities and unsanitary stool disposal are relevant predictors of diarrhoeal disease risk. In the comprehensive model, however, these two WASH predictors also lose statistical significance. All results are shown in Table 2.

In the first model, improved (reference group) as well as unimproved sanitation (1.77, 95% CI 1.14–2.76) facilities were statistically significantly associated with diarrhoea risk, as well as unsanitary disposal of children’s stool (OR 1.54, 95% CI 1.23–1.94). Contrary to expectations, the trend in the ORs for water source suggests that unimproved sources of drinking water as well as surface water may be protective against diarrhoea. With increasing adjustments for non-modifiable characteristics (model 2), susceptibility (model 3) and socio-economic characteristics (model 4), the effect of unsanitary stool disposal becomes non-significant in models 2, 3 and 4. Improved and unimproved sanitation facilities remain statistically significant in models 2 (unimproved, OR 1.98, 95% CI 1.23–3.18) and 3 (unimproved, OR 2.01, 95% CI 1.24–3.24). Distance to water source exceeding 5 minutes from a participant’s home were also associated with increased diarrhoeal risk in models 2 (OR 1.43, 95% CI 1.03–2.00) and 3 (OR 1.40, 95% CI 1.00–1.96).

**Table 2:** Effect of WASH complex and other risk factors on childhood diarrhoea: WASH complex (Model 1) and adjustments for non-modifiable factors (Model 2), susceptibility (Model 3) and socioeconomic characteristics (Model 4).

Risk Factor		Model 1				Model 2						Model 3				Model 4				
		OR	OR (95% CI)		*p* Value *	OR	OR (95% CI)			*p* Value *		OR	OR (95% CI)		*p* Value *	OR	OR (95% CI)			*p* Value *
**WASH**																				
Sanitation	Improved	1.00	--	--	0.04	1.00	--	--		0.03			--	--	0.02		--		--	0.11
	Improved, shared	0.95	0.72	1.26	0.73	0.97	0.73	1.29		0.83		0.97	0.72	1.29	0.83	0.86	0.63		1.16	0.32
	Unimproved	1.77	1.14	2.76	0.01	1.98	1.23	3.18		0.00		2.01	1.24	3.24	0.00	1.63	0.99		2.69	0.05
	Open defecation	1.27	0.90	1.81	0.17	1.26	0.87	1.84		0.22		1.33	0.91	1.94	0.14	1.10	0.73		1.67	0.65
Unsanitary Disposal of Children’s Stool		1.54	1.23	1.94	0.00	1.07	0.83	1.39		0.60		1.07	0.83	1.39	0.59	1.08	0.83		1.40	0.58
Water Source	Piped	1.00			0.19	1.00				0.26					0.26					0.61
	Improved	1.25	0.91	1.70	0.16	1.17	0.84	1.63		0.35		1.15	0.83	1.60	0.40	1.08	0.78		1.51	0.64
	Unimproved	0.93	0.67	1.29	0.66	0.88	0.62	1.24		0.46		0.86	0.61	1.22	0.41	0.90	0.63		1.29	0.57
	Surface	0.84	0.19	3.85	0.83	0.61	0.13	2.92		0.53		0.56	0.12	2.71	0.47	0.56	0.12		2.75	0.48
Distance to Water Source	On premises or delivered	1.00			0.25	1.00				0.09					0.10					0.27
	<5 min	1.02	0.74	1.42	0.90	1.05	0.74	1.47		0.80		1.00	0.71	1.42	0.98	0.91	0.64		1.30	0.62
	≥5 min	1.29	0.94	1.77	0.11	1.43	1.03	2.00		0.04		1.40	1.00	1.96	0.05	1.23	0.87		1.75	0.24
**Non-Modifiable Characteristics**																				
Child Age						0.97	0.96	0.98		0.00		0.99	0.96	1.02	0.39	0.99	0.96		1.02	0.40
Male Sex						1.05	0.84	1.32		0.65		1.04	0.83	1.31	0.74	1.04	0.82		1.31	0.75
Multiple Birth						0.35	0.05	2.66		0.31		0.36	0.05	2.69	0.32	0.35	0.05		2.70	0.32
Region	Ilocos Region					1.00				0.00					0.00					0.00
	Cagayan Valley					0.72	0.33	1.57		0.41		0.69	0.31	1.52	0.35	0.65	0.29		1.43	0.28
	Central Luzon					1.19	0.63	2.26		0.60		1.16	0.61	2.21	0.65	1.17	0.61		2.25	0.63
	Bicol Region					0.51	0.24	1.11		0.09		0.48	0.22	1.04	0.06	0.46	0.21		1.00	0.05
	Western Visayas					1.53	0.80	2.90		0.20		1.47	0.77	2.81	0.24	1.44	0.75		2.76	0.27
	Central Visayas					0.95	0.48	1.91		0.89		0.93	0.46	1.87	0.84	0.87	0.43		1.76	0.70
	Eastern Visayas					1.00	0.49	2.05		1.00		0.95	0.46	1.95	0.88	0.93	0.45		1.94	0.86
	Zamboanga Peninsula					1.11	0.55	2.25		0.77		1.12	0.55	2.27	0.76	1.03	0.50		2.13	0.93
	Northern Mindanao					0.51	0.22	1.21		0.13		0.49	0.21	1.16	0.11	0.48	0.20		1.14	0.10
	Davao Peninsula					0.49	0.21	1.13		0.10		0.48	0.21	1.10	0.08	0.45	0.19		1.04	0.06
	SOCCSKARGEN					2.04	1.06	3.91		0.03		1.98	1.03	3.80	0.04	1.96	1.00		3.83	0.05
	Caraga					0.95	0.47	1.93		0.89		0.90	0.44	1.83	0.77	0.85	0.41		1.74	0.65
	National Capital Region					1.08	0.56	2.08		0.82		1.06	0.55	2.06	0.86	1.09	0.56		2.13	0.80
	Cordillera Admin Region					0.75	0.32	1.71		0.49		0.72	0.31	1.66	0.44	0.71	0.30		1.67	0.44
	ARMM					1.34	0.63	2.82		0.44		1.37	0.64	2.94	0.41	1.92	0.68		5.38	0.22
	CALABARZO					0.87	0.45	1.68		0.67		0.85	0.44	1.66	0.64	0.86	0.44		1.67	0.65
	MIMAROPA					1.10	0.54	2.26		0.79		1.01	0.49	2.09	0.97	0.97	0.47		2.02	0.94
Rural Residence						0.93	0.71	1.22		0.61		0.91	0.69	1.19	0.48	0.86	0.65		1.13	0.28
**Susceptibility**																				
Ever breastfed												0.98	0.64	1.51	0.94	0.92	0.60		1.42	0.71
Vitamin A Supplementation												1.19	0.86	1.64	0.29	1.19	0.87		1.65	0.28
Iron Supplementation												0.93	0.73	1.20	0.59	1.00	0.77		1.29	0.99
Intestinal Parasites Medication												1.21	0.91	1.61	0.20	1.17	0.87		1.56	0.30
Vaccination Index (VI)	Low											1.00			0.00 **	1.00				0.00 **
	Intermediate											0.89	0.36	2.23	0.81	0.91	0.36		2.30	0.85
	High											2.29	1.02	5.14	0.04	2.43	1.07		5.50	0.03
Interaction VI and Child Age	Low VI—Child Age														0.00 **					0.00 **
	Intermediate VI—Child Age											1.03	0.99	1.07	0.17	1.03	0.99		1.07	0.20
	High VI—Child Age											0.97	0.94	1.00	0.10	0.97	0.94		1.01	0.11
**Socioeconomic Characteristics**																				
Maternal Age																0.99		0.97	1.01	0.31
Maternal Education	None or primary																			0.17
	Secondary															0.78		0.58	1.04	0.09
	Higher															0.73		0.50	1.07	0.11
Mother working																1.16		0.91	1.47	0.24
Household Wealth Index	Poorest																			0.28
	Poorer															1.03		0.73	1.45	0.86
	Middle															0.83		0.54	1.27	0.39
	Richer															0.62		0.37	1.05	0.08
	Richest															0.67		0.36	1.24	0.20
Religion	Roman Catholic																			0.68
	Protestant															0.71		0.32	1.57	0.40
	Islam															0.82		0.46	1.46	0.50
	Other															1.10		0.78	1.56	0.60
Number of Household Members																1.00		0.94	1.06	0.96
Number of Under Five Children in Household																1.00		0.84	1.19	0.96
		Nagelkerkes *R*^2^ 0.023				Nagelkerkes *R*^2^ 0.072						Nagelkerkes *R*^2^ 0.085				Nagelkerkes *R*^2^ 0.094				

* *p* values for the significance of variables overall are based on the likelihood ratio test.

The comprehensive model (model 4) revealed two variables as predictive of diarrhoea: region and vaccination index (VI); the interaction between VI and child age was also significant. Moreover, unimproved sanitation facilities remained borderline significant (OR 1.63; 95% CI 0.99–2.69). A high VI was associated with an increased risk of diarrhoea when compared to a low VI (OR 2.43, 95% CI 1.07–5.50). ORs for different regions range from 0.45 (95% CI 0.19–1.04) for Davao Peninsula to 1.96 (95% CI 1.00–3.83) for region XII. Northern Mindanao showed a relatively lower risk of diarrhoea (OR 0.48, 95% CI 0.20–1.14). The explained variance (Nagelkerke’s *R*^2^) was low for all models but increased from 0.023 in model 1 to 0.094 in model 4.

In the sensitivity analysis excluding the vitamin A supplementation variable, unimproved facilities showed a statistically significant effect in all four models. Also, regional effects were more pronounced than in the original model, as was the effect of household wealth.

### 3.2. Qualitative Component

This section describes insights gained with respect to defecation and anal cleansing practices based on FGDs with women in Northern Mindanao. The four urban groups were homogenous in terms of age, while rural groups were relatively heterogeneous. No one refused to participate in the study. Characteristics of all FGDs are presented in Table 3.

**Table 3:** Characteristics of Participants of Focus Group Discussions.

No.	Setting	Number and Sex of Participants			Range
		Female	Male	Total	
1	Rural	73	10	83	19–81
2	Urban	15	0	15	18–39
3	Rural	112	2	114	18–72
4	Urban	10	0	10	35–58
5	Urban	32	0	32	19–73
6	Urban	9	0	9	37–58

**Figure 3:**
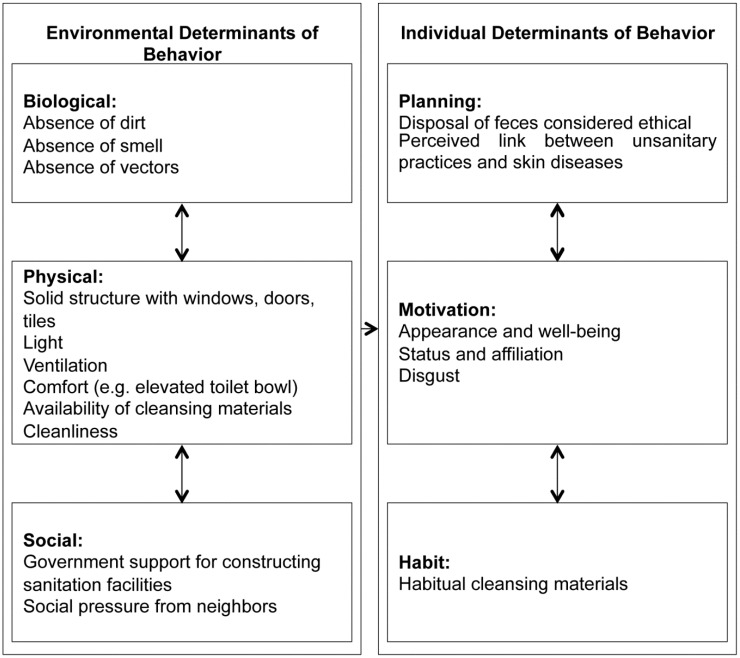
Major Determinants of Defecation and Anal Cleansing Behaviours, as Identified Through Thematic Analysis.

As described under Methods, we structured the findings of thematic analysis according to the framework in Figure 2, focusing on differences between urban and rural settings, as well as insights regarding children; the latter are highlighted in separate sections. First, behaviours related to defecation and anal cleansing are reviewed, followed by environmental and individual factors determining these behaviours. Major determinants of defecation and anal cleansing behaviours are summarised in Figure 3. Findings are based on the data of all six FGDs, with selected citations illustrating these insights.

#### 3.2.1. Defecation and Anal Cleansing Behaviours

Urban settings are generally characterised by higher technical standards, with pour flush and automatic flush toilets being the most common. In terms of superstructure, the urban population predominantly uses elevated toilet bowls, with some participants reporting squatting on in-ground toilet bowls. In contrast, simple latrines and open defecation are common practice in rural settings. In both settings, facilities are often shared with other households. In general, two cleansing behaviours are observed: wiping and washing. Anal washing is the preferred cleansing method for urban and rural groups. In order to perform anal washing water is drawn with a tabo (dipper) out of a bucket or a faucet, and both hands are used for washing.

“*[...] now since we are living in the city, it’s [other cleansing materials] not necessary anymore especially as water is always available*.”

(FGD 2, Urban)

“*You wouldn’t want it [toilet paper] because you prefer water more because it is clean*.”

(FGD 4, Urban)

Water is often not available inside the toilets due to the properties of the facility and lack of piped water on premises. In rural facilities water is usually only available on-site where defecation takes place close to or in a water source. In urban areas, piped water is characterised by its unreliability, making storage inevitable. In both settings facility users are therefore required to ensure that water is available before using the facility.

“*Others just bring water in a pail when they feel the urge*.”

(FGD 1, Rural)

Wet cleansing materials require drying. Towels, old cloth or old underwear are preferred materials for anal wiping, depending on socio-economic status. Among the wipers, there are clear discrepancies between the settings in terms of wiping materials. In rural settings, natural materials such as corn cobs, coconut husks, dried leaves and sticks are used. Fresh leaves were described as slippery and thus not considered adequate for anal cleansing since they fail to soak up faeces. After use materials are burnt or simply left behind and used to cover faeces, where open defecation is practiced. In contrast, using various forms of paper is relatively common in urban areas.

“*When in the middle of the banana plantation, we just use the old banana leaves*.”

(FGD 1, Rural)

“*Especially the urban poor they also use that one [toilet paper], also newspapers. [...] I think they use it because sometimes it is really hard to bring in all the water you need for cleaning*.”

(FGD 6, Urban)

“*[...] usually I also use water, but if there is no water available and I do not want to go out to get water at the faucet I just use the paper*.”

(FGD 6, Urban)

In urban areas, solid cleansing materials are disposed in canisters or garbage bins for waste collection, since participants are concerned about clogging the sewage system.

#### 3.2.2. Environmental Determinants of Behaviour

Environmental determinants of behaviour took up a major part of the discussions, comprising aspects of the biological, physical and social environment (Figure 3). The physical structure of a facility critically influenced defecating behaviour. Respondents in urban and rural areas all valued comfort, privacy, safety and cleanliness. Within the rural groups, privacy was ranked lower than comfort.

“*For me, we used bamboo as wall and it’s really closed. No one can see you when you are inside. So really comfortable*.”

(FGD 1, Rural)

“*The important thing is that there’s that bowl where we can sit down, never mind if someone is there watching us*.”

(FGD 1, Rural)

Comfort is also a consequence of the position of defecation. Elevated toilet bowls for sitting were preferred in private facilities while they were avoided in shared facilities due to possible contamination. Light and ventilation are described as desirable when discussing “the ideal toilet”.

Cleanliness in facilities was emphasised by all groups.

“*There are also others that just like to do squatting position even though it’s supposedly done in sitting position because the toilet bowl is dirty. They are not comfortable.*”

(FGD 2, Urban)

In terms of the social environment, it was noted that the government supports facility construction; however, digging the pit has to be financed by households themselves, often exceeding financial means. In rural communities, social pressure towards improving sanitation is common.

“*Before, when we used the Antipolo type, our neighbour complained because of the awful smell*.”

(FGD 1, Rural)

Biological factors influencing behaviour were mainly dirt and the presence of insects. Elevated positions were preferred due to greater distance from faecal matter.

“*There are lots of skin problems with their kids. And there are a lot of flies and mosquitoes*.”

(FGD 4, Urban)

#### 3.2.3. Environmental Determinants of Behaviour: Insights Related to Children

The lack of water for anal cleansing is a physical constraint to hygiene in schools. The responsibility for water procurement rests with school children themselves. As a consequence, children are frequently absent from school.

“*[…] there was no available water at that time and they were asked to fetch water first from a distant source before they were allowed to use the toilet*.”

(FGD 4, Urban)

The lack of action to improve school sanitation standards was also discussed. Especially the fact that children had to leave school when feeling the urge to defecate was criticised. Public schools were considered to offer higher standards than private schools.

“*[The school] is quite distant from our house but they still want to go home. There was this one time that my kid was really uneasy and he wanted to run home. He failed to reach home and defecated in his pants*.”

(FGD 4, Urban)

“*They have [a] toilet in every classroom. But then, the private schools don’t have the same facilities. The toilets are communal. The public have much better sanitation facilities*.”

(FGD 4, Urban)

#### 3.2.4. Individual Determinants of Behaviour

Based on the discussions individual determinants of behaviour encompass planning, motivation and habit (Figure 3). Habitual mechanisms are clearly influential. Disregarding the inconvenience, people are used to bringing water to facilities.

“*Others still bring water along with them even when they are at the banana plantation area*.”

(FGD 1, Rural)

“*It had already become a habit to bring in water when going to the toilet*.”

(FGD 4, Urban)

Participants revealed a range of motivations for sanitary anal cleansing. Soap is used for its fresh smell, leaving users with a pure feeling and greater confidence in their appearance. On the other hand, being disgusted by faecal matter emerged as a strong motivational driver throughout the FGDs. Societal status was reported as important. In every FGD, participants described a range of “dirty” or unsanitary practices they do not wish to be associated with.

“*It’s really smelly when we don’t use soap for anal washing*.”

(FGD 2, Urban)

“*There is this bushy area in our vicinity, it really smells like shit. I hate it when we pass there. It is where they dispose of all their wastes.*”

(FGD 5, Urban)

“*I have neighbours who defecate in plastic bags or newspapers and throw them in garbage bins. Their kids also, I noticed that they use newspapers for defecating, and the mother just puts the newspaper in the plastic and then in the bin*.”

(FGD 4, Urban)

It was difficult to identify patterns suggesting planned defecation or anal cleansing behaviour with the intention of achieving long-term goals. Diarrhoea was not mentioned in the context of anal cleansing, although the connection was made between “unsanitary” practices and skin problems; concerns about mosquitoes and dogs might also be indicative of planned behaviour. Likewise, covering faeces and disposing of them in a proper way, as pursued by the majority of the population, is considered ethical.

#### 3.2.5. Individual Determinants of Behaviour: Insights Related to Children

Habit appears to be leading the actions of children with children showing a clear preference for using the familiar facilities at home rather than unfamiliar facilities at school. Also, open defecation occurs due to habit.

“*Although our toilet is not that nice, [...] he feels really comfortable using it because it is clean. And we always have water inside the toilet because we have a 6 gallon container*.”

(FGD 4, Urban)

“*They [Ecosan toilets] are not often used by children. [...] They were taught but they don’t like to use it. It is too complicated for them*.”

(FGD 1, Rural)

“*In our community [...], the kids even defecate in an open area and just anywhere*.”

(FGD 6, Urban)

Children generally adopt the anal cleansing practices of adults. One woman reported that her children take a full bath after defecation, a practice taken over from the father.

“*My kids are all naked when they use the toilet. [...] They have their own towel. They use it right after defecating. They will take a bath but not including the head and then wipe themselves*.”

(FGD 4, Urban)

The FGDs with women did not suggest planning or motivation as major drivers of their children’s defecation and anal cleansing behaviours.

## 4. Discussion

### 4.1. Integration of Findings

**Figure 4:**
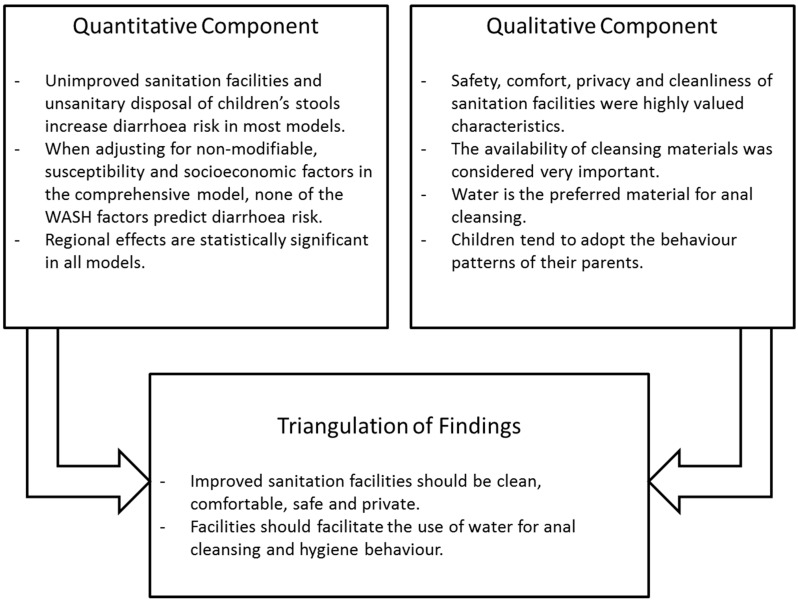
Summary of Key Findings.

Traditionally, quantitative methods are used to describe relationships between sanitation and hygiene behaviours and health outcomes, while qualitative methods are applied to reveal causes and intentions of sanitation and hygiene behaviours. Only a combination of both methods can do justice to the complexity of WASH behaviours [32]. Our study confirms that behavioural and environmental factors interact in determining hygiene behaviour. While the quantitative component suggested that improved sanitation facilities and sanitary disposal of children’s stool are protective against diarrhoeal diseases (even if neither of the two variables showed overall statistical significant in the comprehensive model), the focus groups shed light on the importance of the specific characteristics of hardware for the pursuit of hygienic practices. Figure 4 provides a graphical summary of the main insights derived from the quantitative and qualitative components and their integration; more details are provided in the paragraphs below.

**Water, sanitation and hygiene:** While improved sanitation facilities are protective against diarrhoeal diseases, unimproved facilities increase the risk of acquiring diarrhoea. FGD participants emphasized that a safe, comfortable, private and clean physical structure as well as the availability of water, soap and other cleansing materials are highly relevant for hygiene behaviour. These requirements are often not fulfilled in unimproved facilities, such as pit latrines or hanging toilets. Sharing an improved sanitation facility did not show a statistically significant effect in the comprehensive model. As indicated by the qualitative analysis, insufficient water availability and quantity in the household limits appropriate hygiene behaviours, whether for washing hands with soap or for anal cleansing. Indeed, in the absence of water as preferred cleansing material, a variety of dry materials are used for wiping. The qualitative component of our study moreover confirmed the presence of a range of sanitation facilities and their specific features in local settings of Northern Mindanao. Children tend to adopt the behaviours of adults in their home and community but their defecation and hygiene practices are additionally influenced by low sanitation standards in schools. Both quantitative and qualitative components thus provide insights into the sanitation “hardware” or environmental determinants of hygiene behaviours. The practice of unsanitary stool disposal—a risk factor for diarrhoea—was considered unhygienic and criticised by participants of the FGDs when observed among other community members.

**Non-modifiable characteristics:** As the logistic regression model of childhood diarrhoea is increasingly adjusted, stool disposal loses relevance, while improved and unimproved sanitation facilities as well as distance to water source remain critical predictors for diarrhoeal diseases until the model is adjusted for socio-economic factors. The fact that all WASH factors lose predictive value in the comprehensive model implies that other factors are equally or more relevant in determining disease risk. While we found substantial variation between urban and rural groups in the FGDs (in particular in relation to open defecation and the use of different cleansing materials), such setting-specific differences were not statistically significant in the quantitative analysis. However, region exerts a strong influence on WASH practices and diarrhoea risk, a finding that could not be integrated with the qualitative analysis due to the conduct of FGDs in only one region. This highlights the importance of non-modifiable determinants of hygiene behaviours and points to the need for context-specific intervention designs.

**Susceptibility:** Among the variables to assess a child’s susceptibility to diarrheal diseases, the number of vaccines or vaccine shots a child had received showed a statistically significant effect throughout the analysis. However, a high VI was negatively associated with diarrhoeal disease risk. This suggests that there might be a hidden underlying cause (for example, over-reporting of diarrhoea by those mothers that are more aware of health concerns and therefore more likely to have their children fully immunized), especially as children with a low or intermediate VI showed lower risks. The FGDs highlighted a different aspect of susceptibility: perceived susceptibility to skin diseases as a consequence of unsanitary behaviour with disgust as a major motivator for hygienic behaviours.

**Socio-economic characteristics:** Socio-economic status failed to show an effect in the quantitative analysis. In the FGDs, however, reported possession and use of improved facilities was associated with sanitary anal cleansing. The qualitative analysis moreover showed that women’s and their families’ hygiene practices were greatly influenced by a range of individual determinants, in particular habit, disgust, the wish to be clean and pure and social motivations. Children, even more so than adults, act out of habit and tend to reject facilities or cleansing materials they are not familiar with. An understanding of socio-cultural motivations is therefore critical for the design of appropriate sanitation interventions.

### 4.2. Locating Findings in the Literature

The relatively lower overall significance of the WASH complex as a determinant of diarrhoeal disease risk in our DHS analysis, while surprising, accords with the latest global burden of disease assessment. This found a substantially lower percentage of disability-adjusted life years (DALYs) attributable to water, sanitation and hygiene (2.1% of DALYs for the year 1990 and 0.9% for the year 2010 [3]) than previous assessments (6.8% for the year 1990 [33]; 3.7% for the year 2000 [34]).

The effect of individual WASH variables also merits a more thorough examination. For sanitation, the lack of substantial differences between types of facilities in the main analysis can be partially explained by the specific situation of the Philippines, where sharing improved facilities is common; indeed, the findings of sensitivity analysis 1 accord with expectations. For classifying sanitation facilities, we used the technology-based JMP coding. Therefore, factors that were of particular relevance for the FGD participants such as comfort, privacy or safety could not be considered in the quantitative component. A recent systematic review also found that the strength of evidence pointing towards adverse effects of shared compared to individual facilities was weak and should thus be interpreted with caution and more research is needed under what circumstances shared facilities might be safe [35]. Differences in diarrhoeal disease risk between water sources are limited, in line with the Global Burden of Disease study which found no significantly improved effect of piped water or point-of-use or source water treatment compared with improved water [3]. For excreta disposal, our results are supported by a systematic review showing that sanitary excreta disposal can be more effective in diarrhoea prevention than water quality [36]. For distance to water source, we suspect that accessible and plentiful water encourages better hygiene. Indeed, one systematic review suggests that water quantity interventions are more important than water quality interventions [4], although a more recent systematic review claims the opposite [6]. It has also been suggested that the effectiveness of water quantity is determined by the respective water source [37], an interaction we did not observe.

The determinants of anal cleansing behaviour have rarely been studied to date. We found some evidence that the broad causes of hand washing behaviour [20] also apply to defecation and anal cleansing, whereas we decided that Curtis’ framework [20] was also suitable for analysing the determinants of these elements of hygiene behaviour McMahon and colleagues conducted FGDs in rural Kenya to reveal cleansing practices after defecation. Against previous assumptions about health being a major motivator for hygiene, the study concluded that comfort and avoidance of embarrassment were key motivations [38]. As previously shown, disgust was one of the strongest motivations for pursuing certain defecation and anal cleansing practices. Disgust, a basic human trait protecting us from acquiring disease [39], was evoked by bad smell, dirt and unhygienic practices, suggesting a need for increased emphasis on maintenance of facilities and provision of soap and cleansing materials. Indeed, a Columbian study showed that number of toilets was less important than the provision of soap, toilet paper and clean towels in terms of diarrhoeal disease reduction [40]. Likewise, the provision of sanitation facilities in schools can be effective in terms of reducing absenteeism, especially among girls [41], but appropriate maintenance is essential for their use [42].

### 4.3. Strengths and Limitations

The quantitative component of this study is subject to several limitations. DHS surveys are designed to assess a range of aspects related to child and maternal health, and thus only include a small subset of variables relevant to WASH, with variables such as hand washing before preparing meals not being collected. As self-reported measures of hygiene, variables such as hand washing are frequently subject to over reporting [43]. As proxies for hygiene consciousness, variables assessing hygiene practices remain in any case debated [42]. Also, diarrhoea incidence varies with season, with cross-sectional study designs only providing a snapshot of diarrhoea prevalence at a given point in time [44,45]. Several variables were included as proxies for broader determinants of diarrhoeal disease risk such as access to health care (*i.e.*, intestinal parasite medication) which makes correct interpretation of their effect difficult. Moreover, as WASH variables are relatively crude, measurement misclassification may occur. Crude variables and the resulting misclassification are likely to be the main reasons for the relatively low explanatory power of the models, with low *R*^2^ values not being uncommon in the analysis of routine survey data. It should, however, also be noted that all pseudo *R*^2^ measures, including Nagelkerke’s *R*^2^, must be interpreted with caution, as they are a poor approximation of the true percentage of variance explained. A further pitfall that also might bias our results is that cross-sectional study design such as DHS do not collect information on the use of a facility but its existence. However, the actual use is relevant for determining disease risk, especially in children [46]. Several variables may also be subject to recall bias. In particular, diarrhoea episodes occurring 2–14 days before interview are likely to be underreported while diarrhoea episodes occurring within 48 h before interview might be over reported [47,48]. In the Philippine DHS, these time periods are combined in a single outcome variable, therefore likely direction and magnitude of bias are difficult to determine. Lastly, DHS are cross-sectional in nature and therefore causal inferences cannot be made. Due to all these arguments, the DHS and similar surveys could produce misleading results for the association between WASH factors and health [46]. Nevertheless, due to their sampling strategy, high response rates, as well as high-quality data collection these nationally representative surveys are an excellent data source for complex research questions involving a variety of risk factors. Moreover, by using extensive literature searches to identify all likely determinants of diarrhoea a priori and by including all those available in our analysis, we carefully examined the joint effect of WASH variables and various confounders and competing risk factors.

For the qualitative component of this study we relied on convenience sampling. The impact was noticeable in rural areas: as the rural focus groups were conducted in open areas, the group size grew continuously so that, eventually, 114 participants (FGD 3) and 83 participants (FGD 1) including men (FGD 1: 10 men, FGD 3: 2 men) were present. Imposing formal restrictions on participation was considered inappropriate, as it could have disrupted the trust established within the regular mother classes and with the broader community. The size of these groups is likely to have led to the perspectives of more vocal community members being shared more, while the most vulnerable population segments may not have voiced their views. Nevertheless, participants shared their attitudes in an open manner with discussions mostly taking place among “an inner circle” around the interviewer. All FGDs were characterised by a relaxed atmosphere, with both moderators and participants using humour to overcome shame. While not conducting the analysis in parallel, LMP consulted with EAR throughout the process. Thematic analysis was pursued in a strongly data-driven way. The conceptual framework by Curtis was only applied as a last step to structure findings in a way that facilitates comparability between qualitative studies of different WASH behaviours conducted in different settings and regions. Undoubtedly, previous experiences, social identity and the background of the researchers have influenced data analysis. LMP tried to minimise subjective interpretations by constantly scrutinising conclusions. Indeed, as illustrated by the range of citations, our findings provide a rich picture of the perceptions, concepts and emotions driving defecation and anal cleansing practices among women and, to a lesser extent, their children in Northern Mindanao. Notably, FGDs were conducted with adult women and, as a result, any insights related to children are indirect and are unlikely to show the full spectrum of environmental and individual determinants of children’s defecation and anal cleansing behaviours. Ideally, separate FGDs could have been conducted with children of different age groups, in particular school-age children to generate a more in-depth understanding of the role of sanitation and anal cleansing practices in schools. Given the critical role played by women in relation to WASH technologies and behaviours, many of the direct and indirect insights provided are still likely to show relatively broad applicability.

As a means of structuring the findings of the qualitative analysis, we were specifically looking for a framework that combines individual and environmental and social determinants of hygiene behaviours. Curtis *et al.*’s conceptual model was chosen, as this is based on an 11-country review of formative research findings in support of national-level hand washing promotion programs, and thus has high external validity. While this framework was originally developed with reference to hand washing, previous studies found that broad causes of hand washing behaviours are also applicable to anal cleansing behaviours [20].

Ideally, qualitative and quantitative components would have been conducted in the same location to account for local patterns, and at the same time to account for seasonal variation in diarrhoeal diseases, for example, by combining representative survey data for the region of Northern Mindanao with focus group discussions. Due to lack of resources we had to rely on routine data sources for the quantitative component, and DHS sample sizes for one region only would not have been sufficient. Nevertheless, combining two methodological approaches provides a much more holistic picture of the WASH situation in the region of Northern Mindanao and, to a lesser extent, of the Philippines more generally and has fostered an in-depth understanding that any single component would not have been able to achieve.

## 5. Conclusions

As stated by the Medical Research Council framework [49], formative research is critical in the planning, development and implementation of complex health interventions, such as those to improve WASH. Our study should be considered a first step in the development of appropriate sanitation interventions as part of Fit for School’s school and community activities in the Philippines.

Importantly, health education to encourage hygiene behaviours among children, whether undertaken through mothers at home or in the school environment, can only be effective, where infrastructure and technical facilities allow for these behaviours to be translated into practice. The qualitative component has identified various environmental features of importance in the design and implementation of socio-culturally appropriate sanitation and anal cleansing interventions. It also suggests that children’s and, by extension, their parents’ hygiene habits critically determine whether they will take up an intervention. Beyond habit, relevant motivations for defecation and anal cleansing behaviours include disgust, appearance and well-being and social status. These insights will need to be taken into account in designing locally acceptable sanitation facilities as well as in developing the educational component accompanying these to build relevant skills and to encourage children to exercise correct sanitation and hygiene practices. Moreover, the importance of disgust, in particular, strongly suggests that WASH interventions should not merely focus on hygiene education and sanitation hardware but explicitly consider supply of soap and cleansing materials and comprise regular cleaning and maintenance of facilities. With respect to schools, a critical next step in the development of Fit for School sanitation interventions would be to discuss different options with students, their parents, teachers and other school personnel, considering availability, acceptability, cost and maintenance requirements [50]. As the quantitative component has shown, having improved sanitation facilities is likely to be protective against acquiring diarrhoea. Regional effects are also of predictive value, therefore implementing sanitation and hygiene interventions is likely to require a degree of adaptation. Beyond the Philippines, our study provides detailed insights into anal cleansing behaviours—an aspect of hygiene that has rarely been studied. Research efforts should focus on the impact of different anal cleansing practices on the risk of disease transmission through the faecal-oral route. This is especially relevant in areas lacking reliable water and soap supply for hand washing.
